# All black: a microplastic extraction combined with colour-based analysis allows identification and characterisation of tire wear particles (TWP) in soils

**DOI:** 10.1186/s43591-024-00102-9

**Published:** 2024-10-30

**Authors:** Alexandra Foetisch, Adrian Grunder, Benjamin Kuster, Tobias Stalder, Moritz Bigalke

**Affiliations:** 1 Institute of Geography, University of Bern, Hallerstraβe 12, Bern, 3012 Switzerland; 2 Institute of Applied Geoscience, Technical University of Darmstadt, Schnittspahnstraβe 9, Darmstadt, 64287 Germany

**Keywords:** Microplastic, Tire wear, Optical microscopy, Machine learning, Segmentation, Soil pollution

## Abstract

While tire wear particles (TWP) have been estimated to represent more than 90% of the total microplastic (MP) emitted in European countries and may have environmental health effects, only few data about TWP concentrations and characteristics are available today. The lack of data stems from the fact that no standardized, cost efficient or accessible extraction and identification method is available yet. We present a method allowing the extraction of TWP from soil, performing analysis with a conventional optical microscope and a machine learning approach to identify TWP in soil based on their colour. The lowest size of TWP which could be measured reliably with an acceptable recovery using our experimental set-up was 35 µm. Further improvements would be possible given more advanced technical infrastructure (higher optical magnification and image quality). Our method showed a mean recovery of 85% in the 35–2000 µm particle size range and no blank contamination. We tested for possible interference from charcoal (as another black soil component with similar properties) in the soils and found a reduction of the interference from charcoal by 92% during extraction. We applied our method to a highway adjacent soil at 1 m, 2 m, 5 m, and 10 m and detected TWP in all samples with a tendency to higher concentrations at 1 m and 2 m from the road compared to 10 m from the road. The observed TWP concentrations were in the same order of magnitude as what was previously reported in literature in highway adjacent soils. These results demonstrate the potential of the method to provide quantitative data on the occurrence and characteristics of TWP in the environment. The method can be easily implemented in many labs, and help to address our knowledge gap regarding TWP concentrations in soils.

**Supplementary Information:**

The online version contains supplementary material available at 10.1186/s43591-024-00102-9.

## Introduction

Microplastics (MPs) are distributed globally and have negative consequences on a variety of organisms [1, 2]. MPs in the terrestrial environment have been increasingly studied for more than two decades and it is estimated that a significant part of all the MPs emitted are tire wear particles (TWP) [3–8], originating from the abrasion of vehicles’ tires on the road [9]. Because of their estimated high contribution to the overall MP pollution and their toxic additives [10, 11], there is a urgent need to assess TWP environmental concentrations in soils. However, while there are first data about the concentrations and characteristics of traditional MPs (such as e.g. PE, PP or PET) [12], there are currently mostly model-generated predictions of the amount of TWP emitted in soil [1, 4, 13]. In Switzerland, an estimate for 2018 predicted a tire rubber input of 0.96 ± 0.35 kg capita^−1^ year^−1^ into the natural environment, 4% of which ended up in the soil compartment [5]. Kole et al. [14] predicted the amount of tire material emitted per capita in 13 countries and found a range of 0.23–4.5 kg year^−1^ with an average of 0.81 kg year^−1^. They found a value of 1.1 kg year^−1^ for Germany, which was supported by Juergen Bertling et al. [15], who predicted 1.22 kg year^−1^. However, measurement data to validate these estimations are scarce [16] and the TWP characteristics in soil, such as size and shape, are yet unknown.

Tires are generally composed of approximately 50% natural or synthetic (styrene-butadiene) rubber, 45% softening and hardening materials, and 5% of other additives. However, this composition can vary widely, depending on the desired application of the final product [17]. Despite the fact that they only partly consist of artificial polymers, TWP are still considered MPs [18]. TWP are formed through abrasion of the tire tread on the asphalt, which typically results in TWP becoming incrusted with minerals from the road. The amount and characteristics of TWP emitted depend on a variety of factors ranging from which initial material the tire and the road are made of, to the habits of the driver [1]. TWP produced in simulators were observed to have a minimum size of about 40 nm [19] and a maximum size of around 400 µm [20]. Once formed, TWP can be deposited on the road and be washed out with the road runoff or emitted to the air and transported to other environmental compartments [4]. Regarding roadside soil, TWP can be transported further away than the directly adjacent zone of the road due to air turbulence induced by high speed vehicles, wind, and spray water [4, 21]. When reaching the soil, TWP are suspected to affect soil properties, such as bulk density and water holding capacity, and leach additives, all affecting soil organisms [22].

Most protocols applied to extract MPs from soil include a density separation [23] in combination with oxidation of the natural soil organic matter (SOM) [24] to get clean MP samples. The oxidation of the SOM is often associated with a pre-digestion step using enzymes [25, 26] or other chemicals [27]. The main difference with TWP is that the mineral incrustation at the surface of TWP can increase the particle density from 1.2 g cm^−3^ to 1.5–2.3 g cm^−3^ depending on their size [28] and thus density separation solutions used for MPs might be too low in density to allow full recovery of TWP. However, the majority of TWP was previously found in the 1.3–1.7 g cm^−3^ density fraction [29], and thus the use of e.g. NaI solutions with a density of 1.8 g cm^−3^ might be sufficient to allow a good particle recovery.

The most common identification techniques used for traditional MPs are not suitable for TWP detection. Fourier-transformed infra-red (FTIR) and Raman spectroscopy cannot be applied as TWP are typically opaque to infra-red (IR) light, due to black carbon total absorbance, and do not deliver specific Raman spectra [2, 30]. Attenuated total reflectance Fourier transformed infra-red spectroscopy (ATR-FTIR) can be used to identify TWP but this method is limited by the size of particles it can measure (≥ 500 µm) [31] and is time consuming as single particle measurements are acquired and the crystal has to be cleaned manually after every measurement. Previous studies have reported TWP concentrations in soil ranging from 155 to 64,000 mg/kg using pyrolysis gas chromatrography mass spectrometry (Py-GC–MS) [32], liquid chromatography (LC–MS) [33, 34], thermal extraction desorption gas chromatography (TED-GC) [30] and headspace gas chromatography mass spectrometry (HS-GC–MS) [35] and observed a positive correlation between TWP concentration and the proximity to the road, the traffic density and the brake and acceleration frequency. Mass spectrometry techniques target analytical markers, which can be organic, polymeric or elemental, to assess TWP concentrations in environmental samples [36]. These molecular markers are considered specific to trace TWP, but are prone to leaching and degradation [34], increasing the uncertainty of the results significantly. Polymeric markers are representative of the polymeric fraction of the tires and are potentially more consistent than molecular markers. Rødland et al. [37] used Py-GC–MS to assess the suitability of different polymeric markers and obtained an accuracy of 85–151%, reflecting how inferring the concentration of TWP from an indirect measure is hindered by the high variability of tires initial composition and marker stability. Furthermore, beside the total concentration, the size and shape of MPs are very important to understand their possible toxicity and consequences in soils [38–40], but mass based techniques are not able to deliver information about the particle size and shape distribution. Rausch et al. [41] applied scanning electron microscopy coupled with energy dispersive x-ray spectroscopy (SEM–EDX) with a machine learning algorithm using morpho-textural and elemental information to automatically quantify and characterise TWP in airborne samples. However, for soil, the TWP would first need to be extracted to avoid SOM interferences and concentrate the TWP compared to soil particles. Additionally, MS and SEM–EDX are comparably expensive techniques and need highly trained personnel.

Based on our information, Knight et al. [36] alone have so far performed an individual particle analysis of TWP in soil. They used optical microscopy to quantify TWP down to 50 µm in size and identified them by their colour, their elasticity and their resistance to stress cracking. Despite the size limitation due to manual particle handling, they reported concentrations between 0.6 ± 0.33 and 65 ± 7.36 TWP mL^−1^ wet sediment in roadside drains but did not describe TWP size and shape distributions. These high concentrations indicate an urgent need for an extraction and identification protocol allowing a direct and precise quantification of TWP in different soils and providing morphological information which is crucial to better understand their formation processes, fate, and potential effect on organisms.

In the present study, we aimed to develop a method for the extraction and identification of TWP in soil samples based on their chemical characteristics (extraction) and characteristic black colour (identification), providing information on the particle number, size, and shape using optical microscopy, which is commonly available in many laboratories worldwide. To do so, we (i) developed a method to extract TWP from soils, (ii) evaluated the efficiency of optical microscopy together with a machine learning algorithm to detect TWP based on their black colour and determined the lower size range limit, (iii) assessed the extraction and identification procedure recovery, as well as the method limit of detection (LOD) and quantification (LOQ), (iv) investigated possible interferences in the TWP identification process from SOM and charcoal and (v) applied the extraction and identification method to highway adjacent soil samples and compared our results to previously reported TWP concentrations.

## Material and methods

### Sample materials

Home-made cryo-grinded TWP were prepared by first producing tire chips from 4 different used tires (Bridgestone Potenza S001 225/45R1895Y MO, Continental Winter Contact TS860 195/65R15 91T M.S, Kleber Viaxer 155/65 R13 and Falken Azenis FR510 255/35 ZR20 97Y) using a planer plough. The tires were chosen randomly from a tire recycling facility. The chips were then milled with liquid nitrogen using a ultracentrifugal mill (ZM200, Restch, Germany) and sieved to < 0.3 mm.

Environmental TWP from a highway tunnel dust were collected in the Büttenberg Tunnel (Switzerland, BE). The dust next to the road was sampled into plastic tubes (Corning® 50 mL centrifuge tubes) using a clean white toothbrush.

Charcoal from a bonfire located in Bern surroundings (46°57′25"N, 7°25′22"E) was sampled in three positions according to the centre of the last fire made there (SI1), to obtain charcoal formed at different temperatures. The sampling was done with a stainless steel spatula and the charcoal material was transferred into separate glass vials.

A plastic-free soil sample from a high mountain area (study site n°29 from Scheurer and Bigalke [42]) was used for method validation purposes and one roadside soil was sampled for method application at 4 increasing distance from a highway in Mattstetten (47°01′30N 7°31′03O), Switzerland, during the summer of 2019. The road had a traffic density of 55,000 cars day^−1^ (SARTC, Swiss Automatic Road Traffic Counts). The soils were taken 1 m (MAT1), 2 m (MAT2), 5m (MAT5) and 10 m (MAT10) away from the road and consist of 3 subsamples collected in 0–5 cm depth on a 10 × 10 cm^2^ area and 5 m apart from each other on a transect parallel to the road. Details of the sampling plan and soil characterisation are provided in SI2. The sampling was conducted using a small steel spade, which was cleaned with distilled water after each sampling to avoid cross-contamination. The soils were packed in aluminium trays, dried at 50°C for 24h in a drying oven and stored at room temperature.

### Extraction

**Fig. 1:**
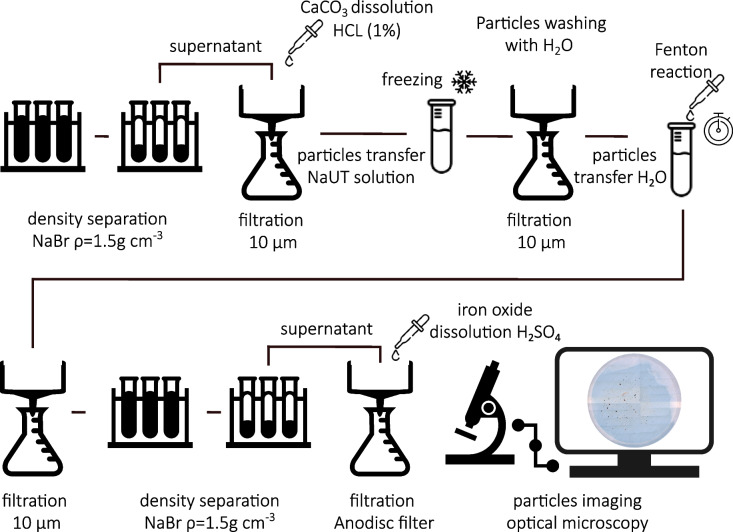
Applied procedure to extract TWP from soil samples

Figure 1 represents the different steps of the TWP extraction method. For each sample, approximately 500 g of soil was sieved to < 2 mm (Retsch, stainless steel, 2 mm) and all visible organic structures (root fragments etc.) were removed with tweezers. As TWP are expected to be mainly in the size fraction < 40 nm to 400 µm (Gustafsson et al*.*, 2008; Kreider et al*.*, 2010), the < 2 mm fraction is expected to contain the majority of TWP.

For TWP analysis, 5 g of dry < 2 mm sieved soil sample and 30 mL NaBr solution (*ρ* = 1.5 g cm^−3^, 9.4:10 NaBr:H_2_O (w/w)) were mixed in a 50 mL plastic tube using a vortex mixer. The samples were sonicated for 10 min and shaken at 100 rpm for 30 min on a shaking plate. To separate the particles > *ρ* = 1.5g cm^−3^ from the ones < *ρ* = 1.5g cm^−3^, the samples were centrifuged at 2500 × g for 30 min. A ‘decanting-aid’ [42] was used to separate the pellet from the supernatant. The supernatant was then transferred to a 10 µm stainless steel filter. The centrifuge tube above the decanting aid was rinsed several times with MilliQ® and the resulting solution was transferred onto the same filter. The decanting aid was then removed from the tube and rinsed onto the filter, as particles might stick to it. Using a vacuum, the NaBr solution was removed, and the particles on the filter were washed with MilliQ®. After releasing vacuum, 10 mL HCl (1%) were added to the filter for 10 min to remove possible CaCO_3_ originating from the soil. The acid was removed by vacuum filtration and the filter was washed with a minimum of 150 mL MilliQ®.

The particles were transferred from the filter into new plastic tube using 25 mL of a mixture of 8% NaOH, 8% urea, 6.5% thiourea (NaUT solution). The NaUT solution was used to predigest the organic structures in the sample before oxidation through the formation of mini crystals [27]. The samples were kept at—20°C for around 40 min (until the first ice crystals are observed) and brought back to room temperature on a shaker at 50 rpm for 30 min. The samples were filtered using a 10 µm stainless steel filter and washed with at least 12 times 30 mL of MilliQ®. This must be done in order to remove all the NaUT solution, as it has a basic pH that could interfere with the following Fenton reaction. From the filters the samples were transferred into new plastic tubes, using as little water as possible (ideally < 5 ml), and 1 mL of Fenton reagent (Fe(II)SO_4_ · 7H_2_O, pH 2.5—3; 0.054 M) and 1 mL of H_2_O_2_ (30%) were added. The Fenton reagent and H_2_O_2_ were added in equal proportions until 15 mL of each was reached. This was done in small steps, always waiting until the reaction was slowing down before adding more reagent. This slow addition of the chemicals was important to avoid a violent reaction and foam formation that could induce a particle loss. In order to keep an optimal temperature for the reaction and to avoid high temperatures (that could affect TWP), the tubes were kept in an ice bath. In case the chemical reaction continued for more than 8h, samples were heated to 50 °C in a water bath [24] to finish the reaction. Next, the samples were transferred again on a 10 µm stainless steel filter and washed with MilliQ® via vacuum filtration.

The samples were transferred back to the tubes using NaBr (ρ = 1.5 g cm^−3^) solution and sonicated, shaken, and centrifuged, as in the first density separation. Next, the supernatant of the samples was filtered on an Anodisc filter (Whatmann® Anodisc inorganic filter, AlO, pore size = 0.2 um, diameter = 13 mm) via vacuum filtration. To dissolve residual iron oxides from the Fenton reaction and avoid filter clogging, 150 µL of H_2_SO_4_ (2M) were added in 50 µL drops to the sample suspension. The filtration apparatus was then gently swirled to mix the added acid with the sample suspension. The inside of the funnel was then rinsed with MilliQ® water to rinse down sample residues adhered to the glass. Once the liquid part of the suspension passed through the filter, the station was de-pressurized. To dissolve all remaining iron oxides, 50 µL of H_2_SO_4_ (2M) was added on the filter directly followed by 15 mL of MilliQ®. These steps, adding 50 µL H_2_SO_4_ (2M) and 15 mL MilliQ®, were repeated until the filter lost its yellowish colour and turned white. Particles still stuck to the inner sides of the glassware were rinsed on to the filter with MilliQ®. The size of the filtration unit was only 13 mm so only the middle of the filter was covered by particles. A detailed list of the materials used for the extraction is provided in SI3.

### Image acquisition, TWP identification and quantification

Sample images were acquired using a Leica M205C microscope equipped with a LED300 RL ring illuminator and mounted with a DMC5400 Leica camera at a magnification of 50x. The light intensity was set at 100% and the exposure was 180 ms. Four high resolution images per filter of 1.5 µm pixel^−1^ were acquired with the Leica software LASX. The plugin Live Image Builder XY was used to record each of the four pictures, as it was not possible to record the whole filter with the plugin due to file size limitations. The four recorded images were manually assembled using GIMP 2.10 [43], rendering a 1.8 GB file. Filter pictures were individually screened and all material obviously corresponding to insect, seeds, roots and leaf fragments were manually covered in white. For the automated picture post-processing the open-source platform Fiji was used. The post-processing included an increase of the exposure and the black levels to enhance the contrast between TWP and background and to remove the filter’s blueish colour [44]. The particles were finally identified by classifying each pixel of the final pictures into a “TWP” or “background” category using the Weka Segmentation [45] plugin in Fiji. Sub-regions of the pictures acquired for blanks, TWP spikes, charcoal spikes and plastic-free soil were used to train machine learning models by marking black particles as potential TWP and other coloured particles as background. The results of the segmentation render a red (TWP) / green (background) binary image. In Fiji, the images were converted into 8 bits and the automatic threshold was applied for the TWP to be black and the background to be white. A watershed function was applied to separate particles which touch each other. For this, the Fiji Adjustable Watershed plugin was used with a watershed tolerance of 3. Finally, the Analyze particles tool of Fiji was used, including particles size of 0 to infinity, to obtain the number and characteristics of all TWP present on a filter. The minimum Feret diameter (mFD) was then used to determine the particle lower size limit detected by the technique and the Feret diameter (FD) was used as the particle size estimation in the data analysis. A detailed example of the individual steps of the image processing is available in SI4. All measurements for individual particles are available in the supplementary files.

### Quality control and quality assurance

The soil sampling was performed wearing non-black clothing. All extraction steps were conducted under a clean hood wearing white cotton lab coat and blue nitrile gloves. All prepared solution were 0.8 µm filtered (Whatman® membranes, 47 mm diameter) and procedural blanks (B) were analysed to assess a potential contamination during the extraction protocol.

For quality control of the extraction and identification method, we choose to use home-made cryo-grinded TWP as spiking material, as no reference material exists yet for TWP. Road dust was not an option because it still contains a larger portion of non-TWP material [46]. Spikes were prepared by suspending 0.1046 g of home-made cryo-grinded TWP in 20 mL of ethanol (≥ 99.8%, Prod.-No 51976, Sigma Aldrich, USA). The suspension was sonicated for 10 min and stirred on a magnetic plate for 30 min. To spike the individual samples, 15 µL of the suspension was collected from the stock solution under constant stirring, deposited onto an Anodisc filter, and imaged with the microscope. After picture acquisition, the spiking material was flushed into a plastic tube using 35 mL of the NaBr *ρ* = 1.5 g cm^−3^ solution. In total, 9 spikes were prepared; 3 of them were directly redeposited on a new Anodisc filter to assess the potential loss of particles during the transfer from the filter to the tube (S0), 3 were used as positive control to assess the extraction and identification recovery (S) and 5 g of the plastic-free soil was added to the last 3 spikes (SPFS) before proceeding to their extraction to assess the recovery rate in a complex matrix.

To determine a possible interference of the charcoal with the TWP during the identification process, 3 charcoal spikes without plastic-free soil (C) and with plastic-free soil (CPFS) were prepared by hand picking charcoal fragments from the 3 sampling position in the bonfire place and depositing them onto an Anodisc filter. After picture acquisition, the charcoal material was flushed into a corning tube using 35 mL of the *ρ* = 1.5 g cm^−3^ NaBr solution.

To assess potential TWP loss during the density separation using a NaBr *ρ* = 1.5 g cm^−3^ solution, environmental TWP from a highway tunnel dust were collected in the Büttenberg Tunnel (Switzerland, BE). 9.9 mg of the collected dust was suspended in 35 mL of NaBr *ρ* = 1.5 g cm^−3^ solution, vortexed for 30 s, shaken for 30 min and centrifuged at 2500*g for 30 min. The particles in the supernatant were separated from the pellet as described in 2.2 using a decanting aid and the supernatant was deposited on polycarbonate filters (Whatman Nucleopore, 47 mm, 0.8 µm pore size, WHA111109). The pellet material was resuspended using MiliQ and deposited on a filter in the same way. The particles were then transferred onto carbon conductive tab fixed on a SEM pin. The morphology and elemental composition of the TWP present in the supernatant were analysed by SEM–EDX (GeminiSEM 450, Zeiss) and compared to the particles in the pellet material. Images were acquired in backscattering mode with a working distance of 8.5 mm and 12 kV.

All samples were processed in triplicate. The description of each sample can be found in Table 1.

**Table 1:** Sample overview and description. The name corresponds to the samples abbreviations used in the present publication

Name	Type of sample	Sample description
**B**	Procedural blank	Only extraction solution; to assess the possible contamination of the samples during extraction
**S0**	Positive control	Spike redeposited on filter without going through the extraction process; to assess the potential particles loss during transfer
**S**	Positive control	Only TW going through the extraction process; to assess the effect of the extraction method on the particles as well as the method recovery rate for TWP
**C**	Interference	Only charcoal going through the extraction process; to determine it can be a source of interference with TWP during the identification method
**PFS**	Matrix blank	Only plastic-free soil; allows to determine the background signal from the soil only
**SPFS**	Positive control in soil	Plastic-free soil and TW; allows to determine the recovery rate of the tire into a complex matrix
**CPFS**	Interference in soil	Plastic-free soil and charcoal; to determine its possible interference with TW identification in a complex matrix
**MAT1**	Environmental	1 m distance from highway; environmental sample
**MAT2**	Environmental	2 m distance from highway; environmental sample
**MAT5**	Environmental	5 m distance from highway; environmental sample
**MAT10**	Environmental	10 m distance from highway; environmental sample

### Data analysis and statistics

All data processing, analysis and plots were performed using R in RStudio [47]. The efficiency of the extraction and identification was assessed by computing the particles recovery rate in different size classes of sample S0, S and SPFS. The effect of the distance from the road was tested by comparing the mean particle number from the triplicates samples at each distance from the road. The comparison was performed by running a non-parametric independent Kruskal–Wallis rank sum test and significant differences were assessed using a Dunn posthoc test using the “FSA” R package. Finally, an estimation of TWP soil contamination was extrapolated from measurement data in particles number per kg dry soil by multiplying the mean particle number of each sample by the initial subsample weight (~ 5g). Yu and Flury [48] showed that the error in MP concentration extrapolation is strongly dependant on the actual concentration of the contaminant, its spatial distribution in the studied matrix, and the volume of matrix extracted. As roadside soils are expected to be highly contaminated and to have a uniform TWP distribution, it can be expected that the sampling error of our concentrations extrapolated from ~ 5g of dry soil samples lies between 5 and 10%.

To investigate the size measurement accuracy of the microscopic set-up and particle recognition, the minimum Feret diameter (mFD) was manually measured on 101 random particles across all the S samples before extraction. The accuracy was calculated by dividing the mFD of the particle as identified by the classification model by the manual mFD measurement and multiplying it by 100. A locally weighted smoothing (LOESS) regression implemented in the R package “ggplot2” was used to help visualize the relation between the particles mFD and the measurement accuracy. Finally, a segmented linear model was fit to the data, to identify from which mFD value the accuracy starts to stabilize, using the *nlsLM* function of the “minpack.lm” [49] R package.

The operational resolution of the method corresponds to a minimum of 1 particle in the processed subsample. Procedural blanks (B) were used to calculate the method limit of detection (LOD) and limit of quantification (LOQ) for soil samples according to a recent study [50] where:

1 $$\text{LOD }= {\text{mean}}_{\text{blank}} + 3*{\text{standard deviation}}_{\text{blank}}$$

2 $$\text{LOQ }= {\text{mean}}_{\text{blank}} + 10*{\text{standard deviation}}_{\text{blank}}$$

## Results and discussion

### Particle detection and recovery

**Fig. 2:**
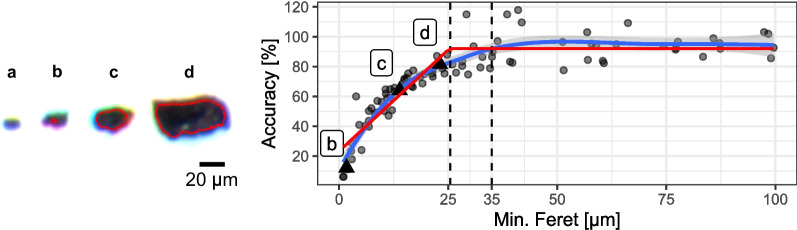
Particle mFD detected by the segmentation model. Left: examples of TWP of increasing sizes (a-d) with the red outline showing their detected perimeter by the segmentation model. Right: Accuracy of the measurement according to the particle mFD. The particles showed on the right are highlighted by triangles in the graph. A LOESS regression is displayed (blue line) to show the general trend of the data. A segmented linear model (red line) shows from which mFD value the accuracy of the measurement stabilises. The dashed lines indicate the position of the particle mFD from which a mean of 90% size accuracy is obtained (25 µm) and the particle mFD threshold applied in this study (35 µm)

The extraction method used here is based on our previous investigations [42] and methods published in the literature [24, 27]. Using the NaUT solution in combination with the Fenton oxidation removed more SOM from the samples and even allowed the analysis of samples with high concentrations of OM. We combined the methods in a way which allowed relatively high sample throughputs for soil samples, by carrying out all steps in centrifuge tubes. With this method we were able to extract batches of 16 samples (one centrifuge load) in 3 days. The analysis of the image segmentation showed that the detection of particles, as well as the accuracy of their measurements, was size dependant (Fig. 2). As the segmentation model was trained to identify black pixels, it was not able to detect the objects where the light diffraction rendered only blue, yellow and red pixels, without having black pixels in the particle centre (Fig. 2a). Particles down to 15 µm mFD were detected but the error in the size measurement was important, as most of the particle surface is diffracting the light (Fig. 2b). The relationship between the measurement accuracy and the detected particle mFD is shown in (Fig. 2, right). The change of point detection function (red line) indicates that the accuracy stabilises around 90% for particles with a mFD > 25 µm. Hence, the experimental set up used in this study allowed for detection and measurement of particle size accurately down to a mFD of 25 µm. For smaller sizes, the measurement accuracy dropped drastically, and the size estimation was not reliable anymore. To lower this detection limit, pictures of a higher magnification could be acquired. However, this would imply dedicating more time for picture acquisition, processing, and segmentation for each sample in instrumental set-up, resulting in a lower sample throughput. Additionally, increasing the magnification will increase the depth of focus and might render sharp images for small objects but blurry for the biggest ones, thus affecting the reliability of the particles size measurements. Time could be saved if (1) pictures were acquired using a motorized xyz microscope stage, which would automatically optimise the focus for each picture and drastically improve the efficiency of picture acquisition and if (2) the segmentation model was run on an analysis cluster with more than 32 GB RAM. With this equipment, increasing the picture magnification and thus lowering the detection limit is feasible. Nonetheless, we consider lower size detection limit of a 25 µm mFD acceptable as it is similar to µFTIR where particles < 20 µm are not consistently detected [51].

**Fig. 3:**
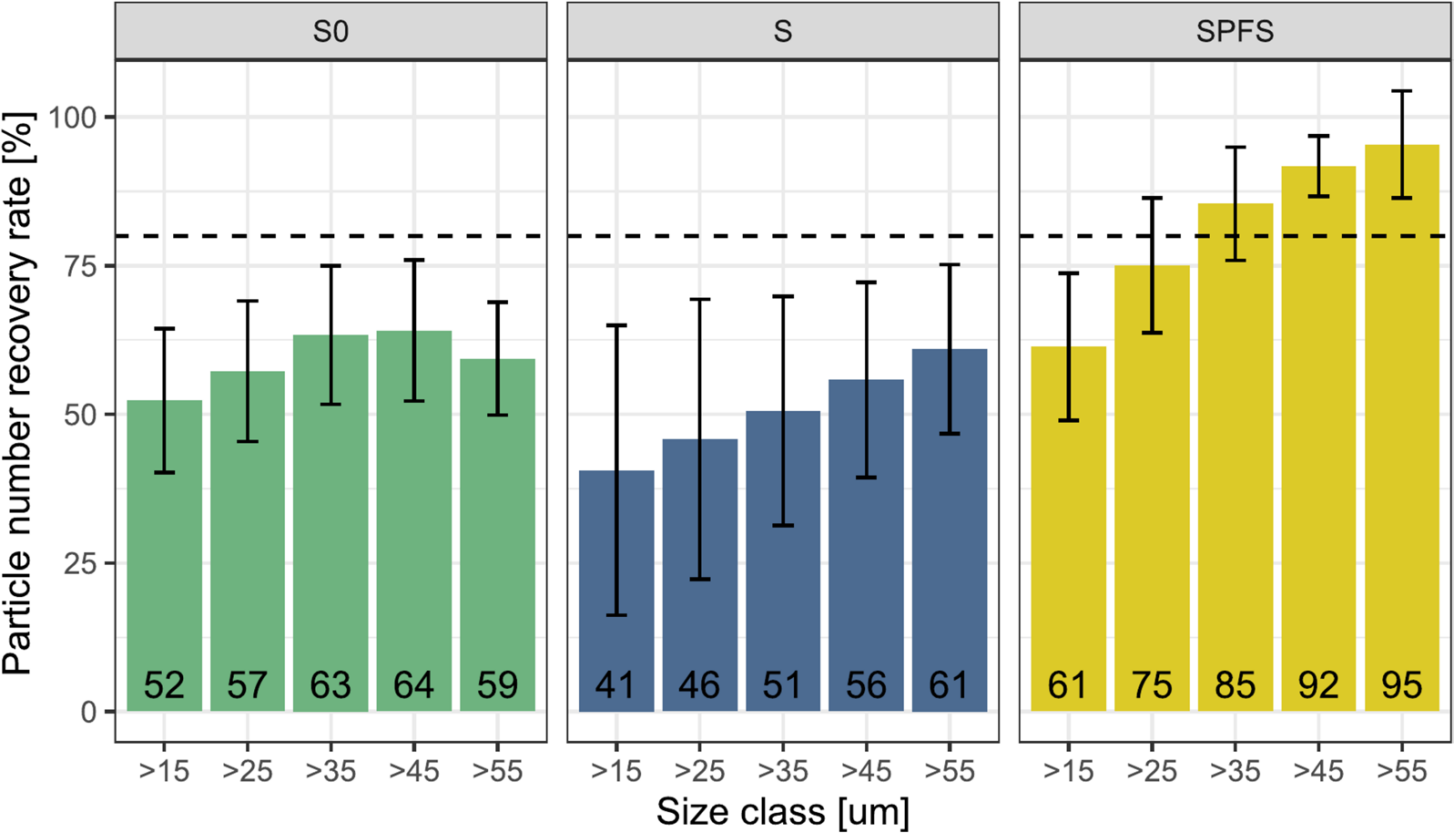
Mean recovery rates with their standard deviation of the TWP spiked samples according to the particle lower size limit (mFD) used for calculating the recovery. S0: spike redeposited on filter without going through the extraction process. S: extraction of TWP alone. SPFS: extraction of plastic-free soil spiked with TWP. The horizontal dashed line highlights the threshold of an 80% recovery rate

The mean recoveries of the different types of spiked samples, according to the lower size range limit, are shown in Fig. 3. Regardless of the minimum mFD particle threshold, the mean recovery rates of spikes without soil (S0 and S) were generally lower than for the spikes extracted in presence of soil (SPFS). Indeed, during sample processing and extraction, we observed that the cryo-grinded TWP were adhering to the plastic tube walls when they were processed without soil. We hypothesized that the adherence was due to a modification of particle surface charges induced by the cryo-grinding and/or sieving processes. In presence of soil (SPFS), however, no particle adhesion was observed. It is most likely that the mechanical forces induced by the shaking steps in presence of soil, together with the chemical interaction of the TWP with the soil constituents prevent their adhesion. A few studies have reported losing particles in their extraction process because of them sticking to their vials’ walls [52, 53], so it might be important that not only the particle properties, but also the matrix is considered. As our method targets the extraction of TWP from soils the SPFS replicates were used to assess the method recovery rates for soil samples. The mean recovery rates of the SPFS samples tended to increase when excluding particles having small mFD from the analysis (Fig. 3), as it was already observed in previous studies [54–56]. This can be due to the fact that smaller particles take longer to migrate in the upper layers of the density separation solution and here only the upper layer of the solution is extracted after centrifugation [57]. In the literature, mean recovery rates for MPs extracted from soil samples can vary between 71% for high density polymers and 93% for low density polymers [58]. To minimize the error in the reported particle concentration values, we chose 80% as a threshold for an acceptable mean recovery rate, setting the particle lower size detection limit to a mFD of 35 µm (recovery rate = 85.4% ± 9.54% standard error) (Fig. 3). This particle size limit is also conservative regarding the accuracy of the automated size measurement, as it is above the estimated lower size limit detected with a 90% size measurement accuracy (Fig. 2, dashed lines).

This recovery rate was obtained by testing the method using cryo-grinded TWP and cryo-grinded TWP can have different properties than environmental TWP, especially in its density. TWP produced from road abrasion are typically incrusted with mineral road material, which can result in particles densities up to 2.3 g cm^−3^ [29, 41, 59]. However, during the extraction of the TWP from the soil, ultrasonication and mechanical shaking are used, which might decrease mineral particle incrustation of the TWP and thus decrease densities. To test for the recovery of environmental TWP we investigated just the density separation using NaBr solution and tunnel dust material. The results of the SEM–EDX analysis on the particles present in the supernatant and in the pellets after the sonication and density separation of the tunnel dust showed that TWP were still present in the pellet (SI5). This indicates that the sonication applied in the extraction protocol does not completely remove the mineral incrustation at the surface of the TWP. Thus, a density > 1.5 g cm^−3^ is needed to extract environmental TWP. As a maximum of TWP was previously found in the 1.3–1.7 g cm^−3^ density fraction [29], this issue can be overcome by replacing NaBr by zinc chloride (ZnCl_2_) or sodium iodide (NaI), which allow for densities of 1.7 and 1.8 g cm^−3^. We recommend the use of NaI for TWP extraction from soils.

### Blanks and interferences

**Fig. 4:**
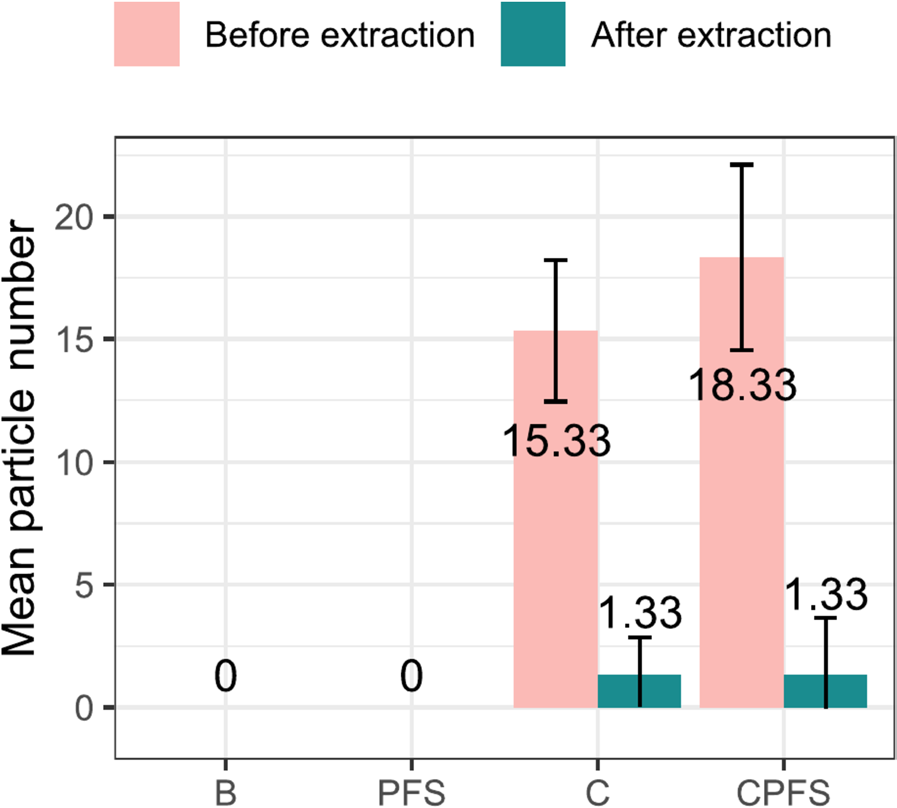
Mean particles number detected in procedural blanks (B, PFS) and interference testing samples (C, CPFS) before and after extraction in the mFD range 35–2000 µm

In the procedural blanks (B) and plastic-free soil (PFS), no particles in the mFD range 35–2000 µm were detected (Fig. 4). This shows that the measures applied to avoid contamination were sufficient and that the chosen plastic-free soil matrix did not cause any interference in the results. Regarding this information, the LOQ and LOD were fixed at the same level as the operational resolution, thus 1 particle per subsample and no blank correction was applied to the environmental samples (MAT1-10).

Charcoal, as a black soil component that can be measured with a similar detection technique [60], may cause interreferences with the TWP detection in soils. Thus, we evaluated the possible interference of TWP detection by charcoal and the diminution of charcoal particles by the extraction method was assessed. The charcoal was not completely eliminated by the extraction process but the charcoal count was reduced by 92%. Despite this strong decrease, an elevated concentration of charcoal present in the soil can interfere with the TWP detection and lead to overestimations of TWP. We assume that black mineral soil components are a minor risk of interference, because mineral particles are efficiently removed with the two density separations during extraction. Also black organic material, which could occur in the samples, is considered a minor threat as almost all organic material is decomposed during the extraction or changes colour during oxidation so that it cannot be mistaken for TWP. Larger organic residues with resistant structures (e.g. root fragments) were removed manually from the image before analysis as described in Sect. "Image acquisition, TWP identification and quantification". 

### Environmental samples

**Fig. 5:**
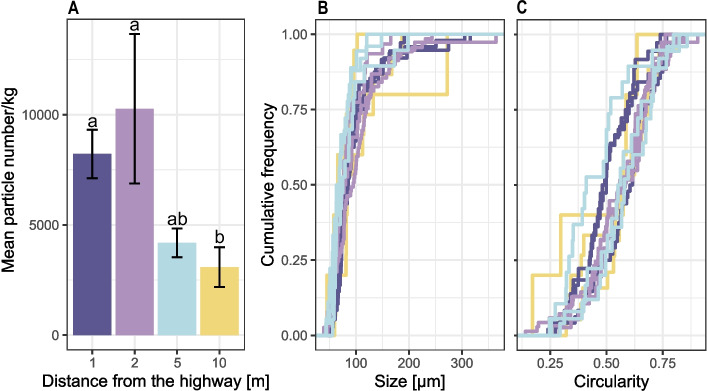
TWP found in environmental samples. **A** Mean particle number kg.^−1^ with standard error. Different letters indicate significant differences between mean particles number (Dunn test, *p*-value < 0.05) (**B**) Cumulative frequency of the particle size using the Feret diameter (FD) as measure for each environmental sample’s triplicate. **C** Cumulative frequency of the particle circularity determined by the image analysis for each environmental sample’s triplicate displayed separately (lines of the same color)

As a proof of concept and to compare the results of the extraction and identification method to previously reported TWP concentrations, soil samples of increasing distances from a highway were extracted and analysed. The extrapolated (SI6) mean particle numbers were 8080 ± 1060, 9107 ± 3235, 4091 ± 624 and 2563 ± 1160 particles kg^−1^ dry soil for MAT1, MAT2, MAT5 and MAT10 respectively (Fig. 5A). There was no significant difference in the particle count at a distance of 1 m, 2 m and 5 m from the road but the particle count at 10 m was significantly lower than the samples closer to the road. To our knowledge, only one study so far reported TWP concentrations in soil samples as particle count [36] but had a particle size limit of 50 µm and did not report their concentrations in particles kg^−1^, which hamper direct concentration comparison. However, they extracted a similar sample amount and had particle concentrations (≈3000 and 14,000 particles kg^−1^ in soils close to the road (Site 3 and 5); calculated from the published data and assuming a soil density of 1 g cm^−3^ with data from [36]) similar to what we found, which shows our detected concentrations are consistent with previous findings. Moreover, the mean particle numbers tended to decrease with increasing distances from the road, as was observed previously for modelled and MS-based data [13, 30, 32, 36, 61]. A rough estimate of the total TWP mass content in each sample was calculated using the model developed by Tanoiri et al. [62] (SI7), giving concentrations of 1.32, 1.94, 0.205 and 0.261 mg kg^−1^ for MAT1, MAT2, MAT5 and MAT10 respectively. These mass estimations are about two orders of magnitude lower than the ones reported from soil samples using styrene-butadiene rubber as a marker (approx. 166 and 71 mg kg-1 at two and five meter distance; own assessment for 0–5 cm depth [30]). The difference may mostly be due to the different site conditions: highways in Switzerland have a hard shoulder, which increase the distance of our sampling points from the actual driving lane by about 3 m. Furthermore, the security lane is regularly cleaned and the road wastewater is collected and treated, which might considerably decrease the TWP concentration in the soil compared to the site described in [30]. Furthermore, the use of NaBr with a low density might also have decreased the particle recovery. Here, we recommend the use of NaI as discussed in Sect. "Particle detection and recovery". Figure 5B and C show the cumulative frequency of the particle FD and circularity, respectively, for each environmental sample triplicate. A circularity equal to 1 indicates a perfect spherical particle while values approaching 0 indicate elongated shapes [44]. MAT5 and MAT10 tend to have a higher proportion of smaller particles than MAT1 and MAT2, which agrees with the assumption that larger particles will be transported over a shorter distance than smaller particles. With a particle upper size limit of 2000 µm, most of the TWP identified in all environmental samples had a FD in the < 200 µm range and the biggest particle was found in MAT2 with a 360 µm FD. Finally, the distance from the road did not seem to have an effect on the circularity of the particles (Fig. 5C) and there was a continuum in particle circularity cumulative frequency, meaning that their shapes were well distributed between elongated and round particles. No correlation between the particle size and circularity was found (SI8).

### Limitations and strengths of the method

As stressed by the UN Plastics Treaty adopted in May 2022, there is an urgent need for a standardized protocol for monitoring TWP in the environment to assess their potential risk, as well as evaluate the effect of future mitigation measures [63]. As an example, California, within the Safe Drinking Water Act, was the first state to agree on MP standard operating procedures [64] to monitor plastic in California’s drinking water, allowing a large scale understanding of MP distribution. Such an agreement also needs to be reached for TWP.

The main limitation of the presented method lies in the uncertainty of the environmental TWP recovery rate. The extraction method applied here was initially developed for non-TW MPs, whose densities rarely exceed 1.5 g/cm^3^, and rendered an excellent recovery rate for cryo-grinded TWP. However, even if most environmental TWP are expected to have a density ≤ 1.7 g/cm^3^ [29], an unknown fraction can be lost during the extraction process. This problem can be solved by replacing the NaBr used for the density separation with NaI solutions with a density of 1.8 g cm^−3^ or higher to minimise the loss of environmental TWP. A second limitation comes from the lack of TWP cross-validation data. If the presence of non TWP black particles was not detected in the matrix blank, this is prone to vary with the origin of soil analysed. A high content of charcoal might interfere with the analysis as charcoal can only be discriminated by around 90% and thus high charcoal counts might cause an overestimation of TWP. To address this, attempts were made to use ATR-FTIR to validate the identification data but, due to the small size of the black particles in our samples, it was not possible to acquire reliable spectra (data not shown). Future work could apply SEM–EDX after fixation of the particles on the filters (to avoid them getting lost in the SEM vacuum) to cross-validate the identification. Finally, only TWP > 35 µm could be identified with our set-up, preventing from the TWP quantification in smaller fractions, which can have the greatest impact on the biota [65]. However, this size limitation is very close to the one of FTIR (~ 20 µm), which is widely applied for MP monitoring in environmental samples.

One of the main advantages of TWP detection using optical microscopy is that it does not require highly trained staff or highly specific lab equipment. Having a simple method facilitates its application on a large scale, as it maximises the number of laboratories and scientific staff taking part in the monitoring effort. The method presented in this study required basic lab and IT equipment (SI3) and can be easily taught to scientific staff. Here, 16 samples required 5 days of technical work for sample and blank extractions and image acquisition and 2 additional days of automated analysis for TWP identification. The results presented above showed that this method was able to detect similar TWP spatial distribution patterns identified by GC–MS and Py-GC–MS [30, 66]. Direct concentration comparison with literature was not yet possible, as the 3 studies on TWP in soil available had different particle size ranges and/or reported their concentrations in mg kg^−1^ [30, 36, 66]. Another considerable advantage is that single-particle analysis is a direct measure and gives information on particle numbers and size and shape distribution, which are not available from MS techniques but are crucial for risk assessment. Furthermore, the direct particle identification by colour is particularly convenient, compared to the detection through analytical markers in the case of TWP: all tyres are black, while the proportion of the target markers can vary broadly in the initial composition of the tires and change over time of the production of the tire, resulting in high uncertainties. Moreover, the concentration of the targeted analytical marker can also change with time when the TWP undergo weathering processes [67], complicating the analysis of environmental TWP, while, to our knowledge, no study showed that weathering factors could affect TWP colour.

## Conclusion

The study presented here showed that our extraction together with optical microscopy coupled to a machine learning classification algorithm was able to accurately detect and measure black particles on white filters down to a mFD of 35 µm, making it a powerful tool for TWP detection in environmental samples. A method recovery of > 85% was achieved using cryo-grinded TWP, with an LOD and LOQ corresponding to the operational resolution of 1 particle. To achieve a similar recovery rate for environmental TWP density solutions from ZnCl_2_ or NaI with a minimum density of 1.7 g cm^−3^ should be used, because of the higher density of environmental TWP. While no TWP were detected in the blank or in the plastic-free soil samples, pieces of charcoal remained after the extraction, indicating a possible interference at high charcoal concentrations. Finally, the analysis of TWP concentration in highway adjacent soil samples showed a similar spatial distribution and order of magnitude to previously reported concentrations. Thus the method presented here is sufficiently accurate and easily implementable in many laboratories, as only basic lab and IT equipment are required, to will allow a large-scale monitoring of TWP in soil samples. However, before its broad application it should be further tested on more different soils to make sure that no unexpected interferences occur with specific soil components, which were not part of the soils tested in this study.

## Supplementary Information

Supplementary Material 1.

Supplementary Material 2.

## Data Availability

The datasets generated and/or analysed during the current study are available in the Zenodo repository: 10.5281/zenodo.13925411.

## Abbreviations

FD: Feret diameter
FTIR: Fourrier transformed infra-red
HS-GC-MS: Headspace gas chromatography mass spectrometry
IR: Infra-red
LC-MS: Liquid chromatography mass spectrometry
LOD: Limit of detection
LOQ: Limit of quantification
mFD: Minimum Feret diameter
MP: Microplastic
MS: Mass spectrometry
NaI: Sodium iodide
NaUT: 8% NaOH, 8% urea, 6.5% thiourea solution
Py-GC-MS: Pyrolysis gas chromatrography mass spectrometry
SEM-EDX: Scanning electron microscopy coupled with energy dispersive x-ray spectroscopy
SOM: Soil organic matter
TED-GC: Thermal extraction desorption gas chromatography
TW: Tire wear
TWP: Tire wear particles
