# Supplementary material for: All black: a microplastic extraction combined with colour-based analysis allows identification and characterisation of tire wear particles (TWP) in soils
Source: Microplast nanoplast. 2024 Oct 30;4(1):25. doi: 10.1186/s43591-024-00102-9 (PMC11525289; doi:10.1186/s43591-024-00102-9)
Supplement: Supplementary file 1 — Supplementary Material 1. [file 43591_2024_102_MOESM1_ESM.docx]

**All black: a microplastic extraction combined with colour-based analysis allows identification and characterisation of tire wear particles (TWP) in soils**

**Alexandra Foetisch^a*^**, Adrian Grunder^a^, Benjamin Kuster^a^, Tobias Stalder^a^, Moritz Bigalke^b^

^a^ Institute of Geography, University of Bern, Hallerstrasse 12, 3012 Bern, Switzerland, ^b^ Institute of Applied Geoscience, Technical University of Darmstadt, Schnittspahnstrasse 9, 64287 Darmstadt, Germany

*correspondence to: [alexandra.foetisch@gmail.com](mailto:alexandra.foetisch@gmail.com)

Supplementary information

# Charcoal sampling positions

To test the interference of charcoal in the TWP identification process, charcoal pieces were collected from a fire place in Bern’s surroundings. The Figure SI 1 shows the 3 positions on which charcoal was collected in a fire place.


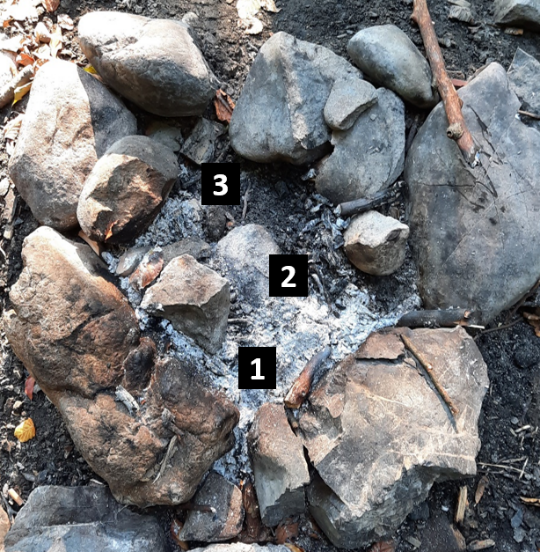


Figure SI 1: Sampling spots of charcoal material from a fireplace in Bern’s surroundings. The charcoal is sampled in 3 areas from the centre (1) of the last fire, to it’s very border (3).

# MAT sampling plan and soil characteristics

The Figure SI 2 illustrate the sampling strategy for the environmental samples located close to a highway in Mattstetten. The 5 g extracted in the study were taken from the pooled samples.


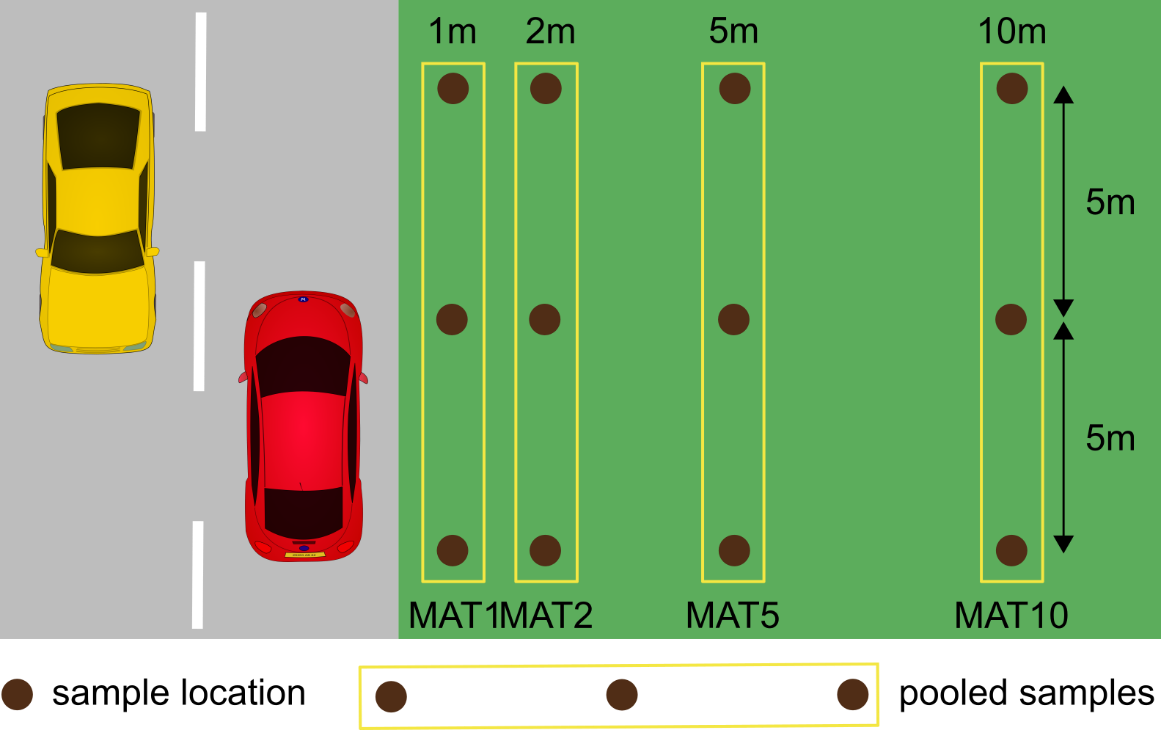


Figure SI 2 Sampling plan for soil expected to be contaminated with tire wear particles. The image is not to scale.

In, the proportion of clay, silt sand and C_org_ are given for the plastic free (PFS) soil and highway soil used in this study.

Table SI 1: Sampling plan for soil expected to be contaminated with tire wear particles. The image is not to scale.

|  | Clay [%] | Silt [%] | Sand [%] | C_org_ [%] | Soil type | localisation | GPS coordinates |
| --- | --- | --- | --- | --- | --- | --- | --- |
| PFS | 7.53 | 49.62 | 42.84 | UP | alluvial | Scuol | 46°47'23.6''N 10°16'22.9'' E |
| MAT | UP | UP | UP | UP | grassland | Mattstetten | 47°01'31.4"N 7°31'07.4"E |

# List of the equipment used for the extraction and identification processes

The following reagents and equipment were used to perform the extraction and identification of TWP at the University of Bern.

**Reagents**

- Iron(II) sulfate heptahydrate (Fe_2_SO_4_ . 7H_2_O), ≥ 99% p.a. ACS, Roth P015.2
- Hydrochloric acid 37 % (HCl), NORMAPUR Reag. Ph. Eur., VWR 20.252.420
- Hydrogen peroxide 30% (H_2_O_2_), Sigma-Aldrich 95321-500 mL
- Sodium bromide (NaBr), 99+% water <1%, Alfa Aesar A10552.0I
- Sulfuric acid 95-97% (H_2_SO_4_), EMSURE Iso for analysis, Merck 1.007.312.511
- MiliQ, Merck ZIQ7000T0
- NaOH >99% (Carl Roth, Art.Nr. 9356.1)
- Urea, Sigma Aldrich, Germany, ≥ 98%
- Thiourea, Merck, Germany, ≥ 98%

**Material and instruments**

- Drying oven
- Mortar
- 5 and 2 mm sieves
- Aluminium containers for dried samples
- Vacuum filtration apparatus for 47 mm membranes
- Vacuum filtration apparatus for 13 mm membranes
- Membranes 0.8 µm, polycarbonate Whatman Nuclepore, 47 mm, Track-Etched Membranes, Sigma-Aldrich WHA111109
- 10 µm stainless steel filter (Rolf Körner GmbH, Werkstoff 1.4401, MW 10 μm, K. 71 μm, S. 41 μm, 200x1400 Mesh)
- Anodisc 25, 0.2 μm, 25 mm, 6809–6022, Whatman membranes, Sigma-Aldrich WHA68096022
- 50ml Centrifuge Tubes (medical grade PolyPropylene) 2*number of sample or glass centrifuge tubes (PYREX(R) CLS9950250-72EA)
- Sonication bath
- Orbital shaker
- Centrifuge
- Decanting-aid
- Pasteur pipette
- Metallic spatula
- Heating blocs
- thermometer
- pH meter
- glass bottles to store solutions
- ice bath
- heating bath
- aluminium foil
- Petri dishes
- Beaker
- Magnetic stirrer and stirring/heating plate
- Laboratory forceps
- Clean hood
- Fume hood
- Microscope
- 32RAM computer
- Fiji free software
- Automatic picture stitching softwar

# Image processing example

The following steps were applied in order to acquire particles count and measurements for each sample described in the linked publication. An example illustrate each step in Figure SI 3.

1. Four automatically stitched pictures were acquired using the Leica LASX Lime image builder xy. The intensity of the light was intentionally kept low to see filter structure and correctly image empty white areas of the filters.
2. The four images are then manually assembled using GIMP2.0 to form a single picture per sample.
3. The picture is cropped to remove the black and blue background originating from the microscope settings.
4. zoomed in from picture showed in 3 (yellow rectangle) is shown to allow a better visualisation of the processing effects
5. Obvious non-TWP are manually covered in white
6. The black levels and the exposure are increased in the whole picture (The following macro commands were used to automate the processing of all pictures:

run("Window/Level...");

setMinAndMax(19, 133);

1. The segmentation of the model is then applied to the whole picture. As this analysis requires a lot of computation power, a high RAM memory is needed to process a picture at once. However, this issue could be overcome by a script allowing to split the picture in tiles, which are then classified one after the other, and merging each tile classification to form the entire result. THe output of the Weka segmentation plugin is a green/red binary image.
2. The image is converted to 8bit and the threshold automatically adjusted. A watershed function is applied using the Adjustable Watershed plugin in Fiji setting the tolerance to 3.
3. The “analyse particles” Fiji tool is finally applied to quantify and measure all particles identified by the model.


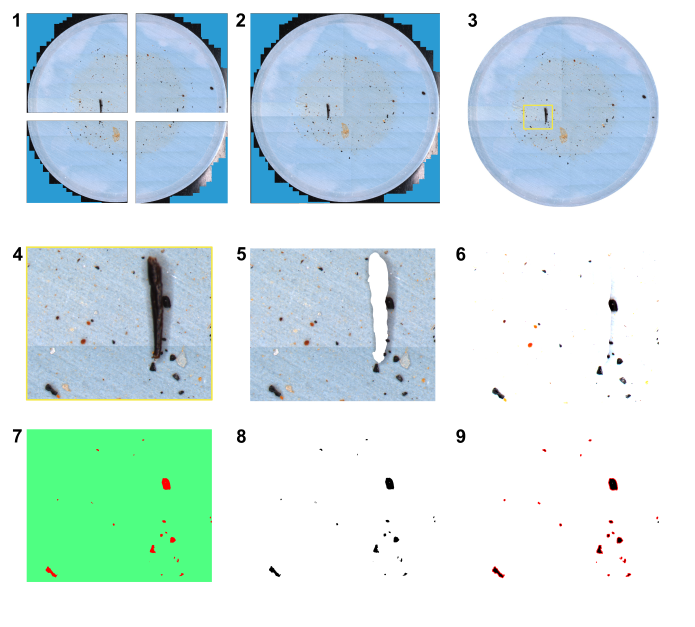


Figure SI 3: Illustration of the picture acquisition and processing steps. The numbers correspond to the numbered items in the text.

# SEM-EDX analysis of highway tunnel dust

Images and EDX spectra acquired on the tunnel dust after density separation with sodium bromide in the supernatant showed the excepted presence of TWP, with a typical elemental composition of C, O, Fe, Na, Mg, Al, Si and Ca (Rausch et al., 2022) (Figure SI 4). Even if there were far less numerous (quantification not available), the same particles could also be detected in the pellet material, indicating the combination of ultrasonication and density separation with NaBr do not allow to isolate the whole range of TWP densities.


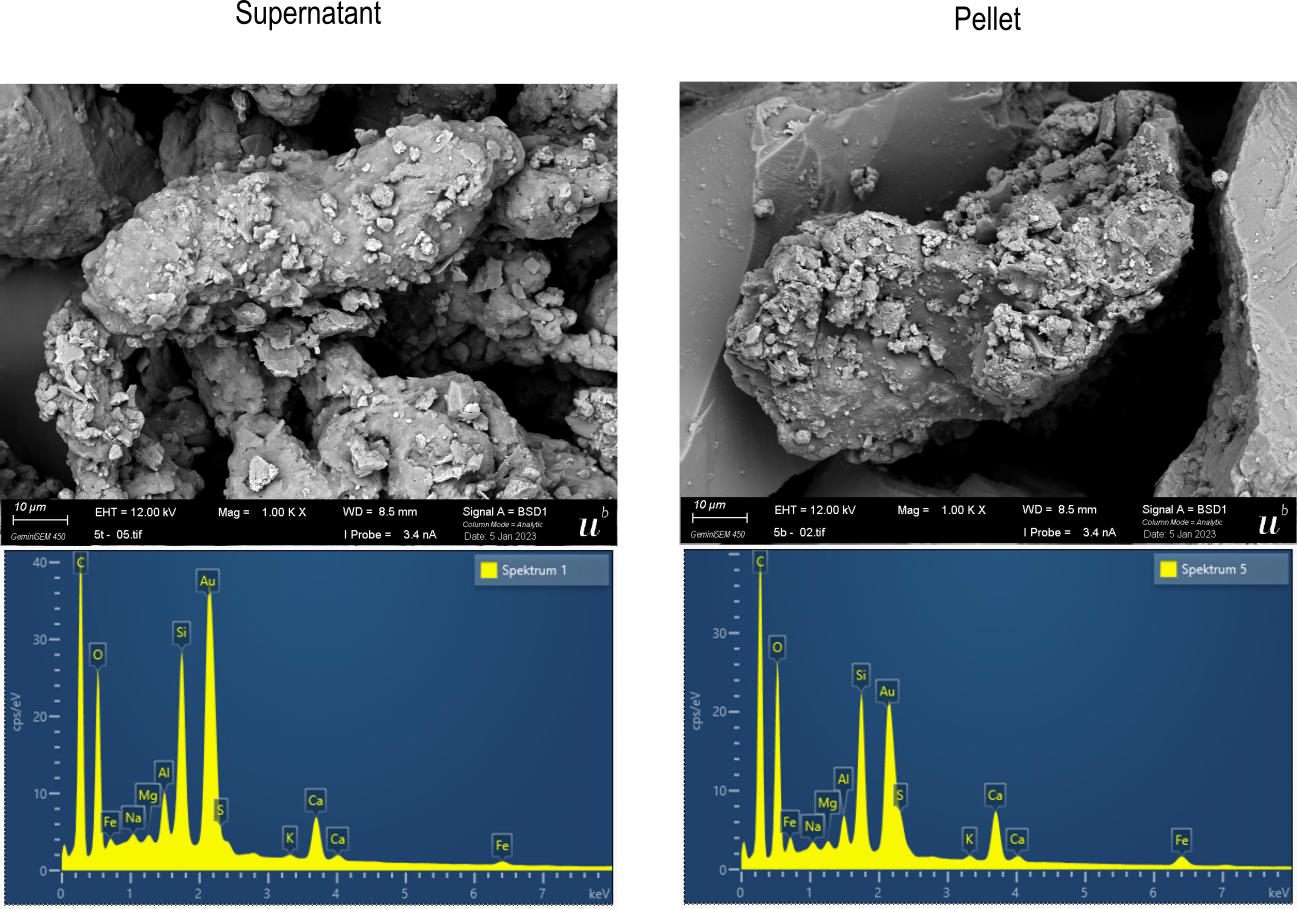


Figure SI 4: Representative example of SEM-EDX analysis of particles found in the supernatant and in the pellet after density separation using sodium bromide (NaBr) salt of a 1.5 g/cm^3^ density.

# Extrapolation of TWP concentrations

The concentration of TWP found in the soil samples were extrapolated to kg^-1^ dry soil to facilitate concentrations comparison. The extrapolated concentrations were calculated as follow:

TWP number [kg-1 dry weight] = $\frac{number of TRWP found}{sample dry weight [g]}$ * 1000 (1)

The mean and standard deviation of the TWP number [kg-1 dry weight] were then calculated for each distance. Raw data and results are presented in Table SI 2.

Table SI 2: Number of particles found in the environmental samples and their extrapolation to kg^-1^ dry soil

| Distance | Number TWP found | Sample dry weight [g] | TWP number  [kg^-1^ dry weight] | Mean TWP number  [kg^-1^ dry weight] | Standard deviation TWP number  [kg^-1^ dry weight] |
| --- | --- | --- | --- | --- | --- |
| MAT1 | 48 | 5.015 | 9571.286 | 8080.427 | 1059.822 |
|  | 38 | 5.088 | 7468.553 |  |  |
|  | 36 | 4.999 | 7201.44 |  |  |
| MAT2 | 69 | 5.070 | 13609.47 | 9106.606 | 3234.823 |
|  | 38 | 5.030 | 7554.672 |  |  |
|  | 31 | 5.036 | 6155.679 |  |  |
| MAT5 | 25 | 5.035 | 4965.243 | 4091.15 | 623.8617 |
|  | 19 | 5.056 | 3757.911 |  |  |
|  | 18 | 5.070 | 3550.296 |  |  |
| MAT10 | 19 | 5.059 | 3755.683 | 2562.835 | 1160.34 |
|  | 15 | 5.098 | 2942.33 |  |  |
|  | 5 | 5.048 | 990.4913 |  |  |

# Estimation TWP mass content in soil samples

The total TWP mass each highway adjacent soil samples was calculated using the model previously developed by (Tanoiri et al., 2021):

$Estimated TWP mass =\frac{4}{3}\left( \frac{Feret}{2} \right)\left( \frac{MinFeret}{2} \right)\left( \frac{0.372*MinFeret}{2} \right)\pi\rho$ (2)

Where Feret and MinFeret correspond to the longest and shortest dimensions of an ellipse fitted on the particle and ρ = 1.2 g/cm^3^ is the estimated density of tire wear(Degaffe and Turner, 2011). The calculated mass concentration of the highway soil samples had a similar variation pattern as the one observed for particles number, where the concentration was higher closer to the road (Figure SI 5)


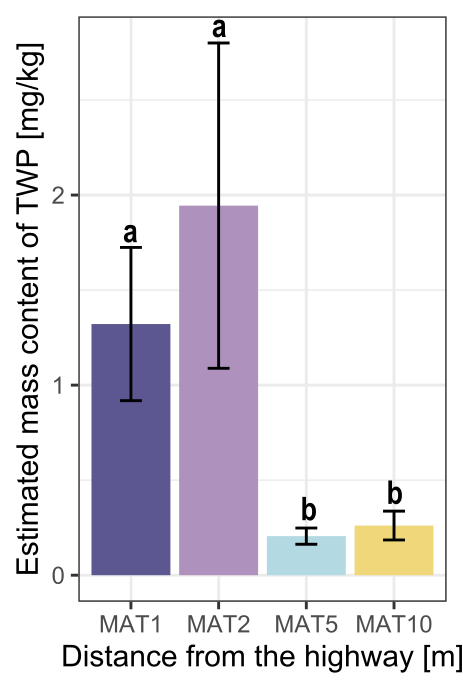


Figure SI 5: Mean TWP mass mg* kg^-1^ with standard error. Different letters indicate significant differences between mean mass content for each distance to the road (Dunn test, p-value <0.05.

# Correlation of particle size and circularity

The Figure SI 6 shows the size of the TWP identified in the environmental samples in relation with their circularity. A circularity value of 1 indicates a perfect sphere, while a value of 0 indicate an elongated shape, such as a fibre. Here, no correlation could be observed, indicating that TWP have all kind of shapes and they are not related to their size.


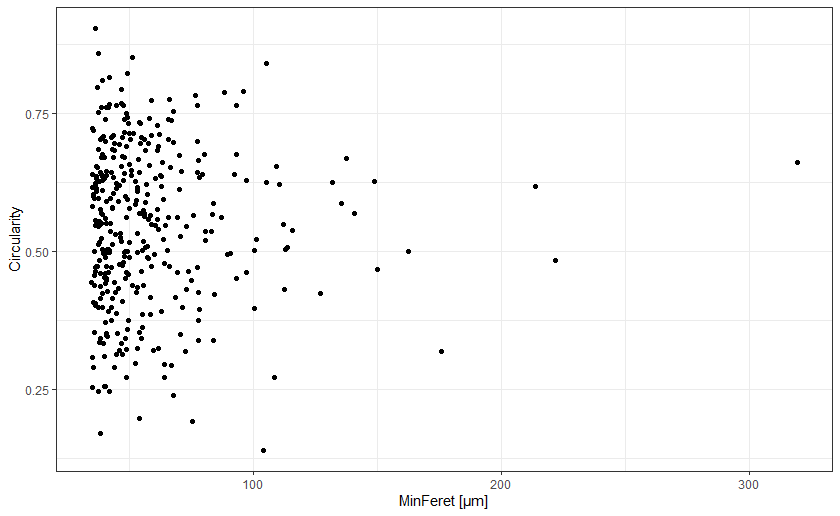


Figure SI 6: TWP particles size (MinFeret) according to their circularity
